# CuPc: Effects of its Doping and a Study of Its Organic-Semiconducting Properties for Application in Flexible Devices

**DOI:** 10.3390/ma12030434

**Published:** 2019-01-31

**Authors:** Mariel Leyva Esqueda, María Elena Sánchez Vergara, José Ramón Álvarez Bada, Roberto Salcedo

**Affiliations:** 1 Facultad de Ingeniería, Universidad Anáhuac México Norte, Avenida Universidad Anáhuac 46, Col. Lomas Anáhuac, Huixquilucan 52786, Estado de México, Mexico; mariel.leyvaes@anahuac.mx (M.L.E.); ramon.alvarez@anahuac.mx (J.R.Á.B.); 2 Instituto de Química, Universidad Nacional Autónoma de México, Circuito Exterior S/N, Ciudad Universitaria, Coyoacán 04510, Ciudad de México, Mexico; salcevitch@gmail.com

**Keywords:** organic semiconductors, thin films, flexible electronics, optoelectronic devices

## Abstract

This study refers to the doping of organic semiconductors by a simple reaction between copper phthalocyanine and tetrathiafulvalene or tetracyanoquinodimethane. The semiconductor films of copper phthalocyanine, doped with tetrathiafulvalene donor (CuPc-TTF) and tetracyanoquinodimethane acceptor (CuPc-TCNQ) on different substrates, were prepared by vacuum evaporation. The structure and morphology of the semiconductor films were studied with infrared (IR) spectroscopy, X-ray diffraction (XRD), and scanning electron microscopy (SEM). The absorption spectra for CuPc-TTF, recorded in the 200–900 nm UV–vis region for the deposited films, showed two peaks: a high energy peak, around 613 nm, and a second one, around 695 nm, with both peaks corresponding to the Q-band transition of the CuPcs. From the spectra, it can also be seen that CuPc-TTF has a B-band at around 330 nm and has a bandgap of approximately 1.4 eV. The B-band in the CuPc-TCNQ spectrum is quite similar to that of CuPc-TTF; on the other hand, CuPc-TCNQ does not include a Q-band in its spectrum and its bandgap value is of approximately 1.6 eV. The experimental optical bandgaps were compared to the ones calculated through density functional theory (DFT). In order to prove the effect of dopants in the phthalocyanine semiconductor, simple devices were manufactured and their electric behaviors were evaluated. Devices constituted by the donor-acceptor active layer and by the hollow, electronic-transport selective layers, were deposited on rigid and flexible indium tin oxide (ITO) substrates by the vacuum sublimation method. The current–voltage characteristics of the investigated structures, measured in darkness and under illumination, show current density values of around 10 A/cm^2^ for the structure based on a mixed-PET layer and values of 3 A/cm^2^ for the stacked-glass layered structure. The electrical properties of the devices, such as carrier mobility (μ) were obtained from the J–V characteristics. The mobility values of the devices on glass were between 1.59 × 10^9^ and 3.94 × 10^10^ cm^2^/(V·s), whereas the values of the devices on PET were between 1.84 × 10^9^ and 4.51 × 10^9^ cm^2^/(V·s). The different behaviors of the rigid and flexible devices is mainly due to the effect of the substrate.

## 1. Introduction

In recent decades, increases in the use of fossil fuels have caused serious environmental problems, releasing toxic compounds and greenhouse gases responsible for air pollution as well as natural resource depletion and global warming. These problems, compounded by growing world population and energy demand, have led the decades-long interest in developing and adopting alternative energy technologies [1]. Technological development and innovation in green energy sources, mainly based on solar energy, have become important and necessary for environmental sustainability [2]. Solar energy is a clean, renewable, and accessible resource [3], as the amount of solar radiation that reaches the terrestrial surface in one hour is higher than the annual world energy consumption [4]. On the other hand, significant challenges remain regarding the efficiency and regularity with which solar energy can be harnessed. New developments in solar energy collection could bring environmental, social, and financial benefits [5,6]. The diversity of technologies for solar-energy production is considerable and includes photovoltaic cells that can be manufactured from inorganic compounds, such as gallium arsenide, cadmium sulfide, and silicon. However, these technologies require expensive equipment and generate highly-polluting residues [7,8,9]. A viable strategy to generate sustainable energy and reduce some of the aforementioned environmental problems should consider the replacement of silicon and inorganic semiconductors through the development of organic semiconductors that permit new approaches for the production of photovoltaic devices [10].

Optoelectronic devices based on organic semiconductors, such as organic photovoltaic cells (OPCs), require stable p- and n-type organic semiconductors with excellent properties. Additionally, it is desirable that these organic semiconductors be easily synthesized; the chemical compounds involved during their manufacturing process should be less toxic for the environment and stable during their whole life cycles. Organic semiconductors, which combine the advantages of plastics (solubility, mechanical strength, modifiable physical-chemical parameters) with electrical conductivities typical of metals, enable implementation of organic devices. Organic electronic products have a large market potential; nevertheless, the implementation of commercial applications of conductive macromolecules still requires more advanced research and improvements in manufacturing technology. A persistent problem regarding the use of organic compounds as semiconductors is the relatively low conversion efficiency of incident radiation to electron flow (e.g., compared to bandgap irradiation of the substrate inorganic semiconductor). This can be attributed to the low conversion efficiency of excitons to separate electron–hole pairs and to the low mobility and rapid trapping of carriers in the sensitizer films. Phthalocyanines (Pcs) show a tendency toward aggregation and polymerization in the solid state. This could lead to a larger orbital overlap between chromophores and improve photogeneration and charge-carrier transport. These organic molecules have found potential applications in numerous fields, such as alternative energy (including photovoltaic cells), gas sensors, organic light emitting diodes, medical applications, etc. The explanation is to be found in their similarities to natural systems (porphyrins, hemoglobin and chlorophyll), that form the basis of many biologically important systems. Unlike these biological compounds, Pcs are completely synthetic compounds and can coordinate in their central cavity more than 70 ions, such as hydrogen or metallic ions, becoming metallophthalocyanines (MPcs), with good thermal and thermo-oxidative stabilities at temperatures beyond 500 °C, and which are thus of great interest for the preparation of thermoset films [11]. MPcs are aromatic plane macrocycles constituted by four units of isoindole and united through the positions 1 and 3 by aza bridges. They are strong absorbers in the spectrum range between 600 and 700 nm, which explains their blue to green-blue color range. The basic skeleton of MPcs is integrated by a π delocalized cloud of 42 electrons over a set of 40 atoms, of which 32 are carbon and 8 are nitrogen. As occurs with porphyrins, electronic delocalization takes place in the interior ring, so they can be considered heteroannulenes formed by 16 atoms and 18 π electrons, the isoindolic units keeping their benzenoid character. Based on the 12 principles of green chemistry introduced by P.T. Anastas and J.C. Warner [12], MPcs can be described as sustainable organic semiconductors in the context of green chemistry. They are produced from cyclotetramerization reactions of different precursors using routes of synthesis that do not generate toxic waste [5], that take full advantage of all the compounds used in doping processes, and involve low-toxicity substances, such as ethanol or methanol [13,14,15,16,17,18,19,20,21]. At the end of their life cycles, they can be solubilized and degraded through the introduction of substituents both in benzene rings as well as coordinated to the central metal. 

This work is about the doping of organic semiconductor through the reaction between copper phthalocyanine (CuPc) and (i) 7,7,8,8-tetracyanoquinodimethane (TCNQ) acceptor and (ii) tetrathiafulvalene (TTF) donor. CuPc is one of the best candidates for optoelectronic device fabrication, because of its semiconducting character when doped [22,23,24]. Dopants were chosen according to their charge transfer properties, optic and electronic behavior, low dimensionality and low heat generation [25]. CuPc is an acceptor moiety for TTF-σ-acceptor [26] and it has been demonstrated that doping p-type semiconductors such as CuPc with TCNQ can turn them into n-type semiconductors [25,27]. Although p-type doping has been tested in many combinations of electron-acceptor molecules, n-type doping has not been as widely studied in organic semiconductors, given the limited availability of strongly-reducing molecules [28]. In this study, the p/n behavior of CuPc in the presence of TTF and TCNQ, in order to determine the doping capacity of these compounds and the feasibility of their use in ambipolar devices, with both p-type and n-type charge transfer. 

The doped material was deposited in thin-film form, which and then morphologically and structurally characterized; its optical and electrical properties were also evaluated. As a sandwich-like configuration is frequently used in the preparation of thin-film devices, structures with this configuration were fabricated by vacuum thermal evaporation. Transport characteristics of organic sandwich-like devices were studied from I–V measurements. In the design of photovoltaic devices, a key element is the transparent conductor electrode, particularly its optical and electrical properties. For this purpose, ITO (In_2_O_3_·(SnO_2_)_x_) is used as a transparent electrode; it functions as an anode or hole injector. Silver electrodes, functioning as cathodes or electron injectors, were also deposited. For optoelectronic device applications, it is necessary that the organic thin film which is in contact with the ITO has an adequate adherence to the ITO film, as well as to the substrate. The adherence to the substrate will not only enhance the dimensional stability of the film, but will also promote efficient charge transport along the whole device. The effects of glass and PET substrates in the manufacture of the devices and their I–V behavior were also studied. The use of PET as a substrate is proposed as a potentially innovative flexible electronics application for this type of devices. Flexible electronics can be classified as an emergent technology whose applications could lead to ultrathin, adaptable devices and products.

## 2. Materials and Methods

### 2.1. General Remarks

CuPc (C_32_H_16_CuN_8_), TTF (C_6_H_4_S_4_), and TCNQ (C_12_H_4_N_4_) were used in this study to obtain CuPc-doped semiconductors; they were purchased from Sigma-Aldrich (Saint Louis, MO, USA) and required no further purification. A series of organic semiconductors were doped by a simple reaction in absolute ethanol between CuPc and TTF/TCNQ in conventionally heated Monowave 50 reactor (Anton Paar GmbH, Graz, Austria) with a pressure sensor. The reactor was operated with a borosilicate glass vial and manually closed by a cover with an integrated pressure (0–20 bar) and temperature sensor. 576 mg (1 mmol) of CuPc were added to 51 mg (0.25 mmol) of TTF or 51 mg (0.25 mmol) of TCNQ, respectively, and dissolved in ethanol. They were kept in the reactor for 30 min and cooled by lowering the pressure and temperature of the system; the doped semiconductor was filtered, washed and dried in vacuum. Both p-type and n-type CuPc doping were carried out. The formation of molecular systems based on both CuPc types (electron donor and acceptor) permits the generation of states with charge separation of large mean lifetimes for applications such as active elements in photovoltaic devices [13]. Melting points were obtained from Sigma-Aldrich certificates, while decomposition points were obtained on a Melt-Temp II apparatus (Thermo Fisher Scientific Inc., Waltham, MA, USA). In order to verify the main functional groups of the organic compounds, IR-spectroscopy analysis was performed on a Nicolet iS5-FT spectrometer (Thermo Fisher Scientific Inc.) on a wavelength range of 4000 to 500 cm^−1^ using KBr pellets. The pellets were fabricated in several steps: first, the doped semiconductors were mixed with pure, dry KBr in an agate mortar until a fine powder was obtained. The powder was put in a sample holder and then introduced into a hand-operated press, whose compression transformed the powder into pellets.

### 2.2. Thin Film Deposition and Characterization

Thin films were deposited simultaneously on Corning glass, quartz, and monocrystalline silicon wafers. To guarantee an accurate performance, all substrates were cleansed under an ultrasonic process using organic solvents (chloroform, methanol, and acetone) and dried in vacuum. The organic semiconductors were deposited by sublimation on a vacuum chamber. Vacuum was accomplished through two pumps: a mechanical pump that generated an initial pressure of 10^−3^ Torr and a turbo-molecular pump that generated the final deposition pressure of 10^−10^ Torr. The deposition process involved an evaporation equipment with molybdenum and tantalum boats. The evaporation rate (0.3 Å/s), temperature (298 K) and pressure (1 × 10^−5^ Torr) in the vacuum chamber were the same for all the deposition processes. During the deposition processes, the films’ thicknesses were evaluated using a high-resolution thickness monitor with quartz sensor SISMONI-1C-3mHz. The TTF-doped CuPc film had a thickness of 70 nm, while the TCNQ-doped film had a thickness of 66 nm. Thickness was the same for the three substrates used in each material’s deposition. IR spectroscopy was carried out in order to determine if the thermal evaporation produced chemical changes and to establish the presence of the representative functional groups in doped CuPc. During thin-film deposition, stress concentration may occur within the material, which causes slight displacements in the spectrum signals, though no chemical degradation of the material would be expected. IR spectroscopy was also carried out with the purpose of detecting thin-film impurities that could affect charge transport in the material. FT-IR analysis of the infrared absorption spectrum was carried out with a Nicolet iS5-FT spectrometer (Thermo Fisher Scientific Inc.) on the silicon wafers. X-ray diffraction analysis was performed in quartz substrates with the θ–2θ technique using a Bragg–Brentano geometry with a Bruker, D8 Avance diffractometer (Bruker Corporation, Billerica, MA, USA) and working with CuK-α (λ = 0.15405 nm) radiation. For SEM, a Zeiss EVO MA 10 scanning electron microscope (Carl Zeiss AG, Oberkochen, Germany) was operated at 3 kV voltage and a focal distance of 13.5 mm, using thin films on a quartz substrate. The films built on quartz substrates were used to measure optical absorption with a Unicam spectrophotometer, model UV300 (Thermo Fisher Scientific Inc, Waltham, MA, USA), in the wavelength range of 200–1100 nm. 

### 2.3. Theoretical Method

DFT calculations were carried out in order to find the bandgap of the doped semiconductors and compared with the experimental bandgaps. The geometries were optimized using a pure DFT (density functional theory) method for energy evaluations, applying Becke’s gradient corrections [29] for exchange and Perdew–Wang’s gradient for correlation [30]. This is the scheme for the B3PW91 method that forms part of the Gaussian 09 Package [31]. All calculations were performed using the 6-31G** basis set. Frequency calculations were carried out at the same level in order to confirm that the optimized structures corresponded to a minimum of the potential surfaces.

### 2.4. Devices Assembly and Characterization

**Figure 1:**
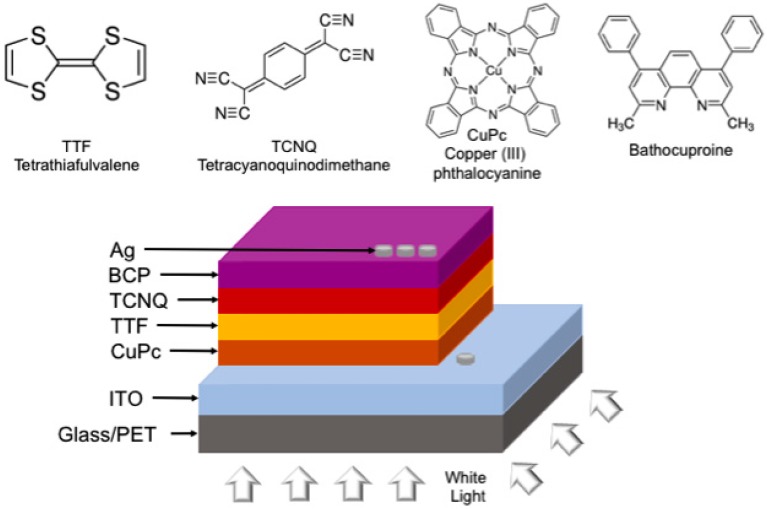
Structure of the devices.

Simple devices were prepared from the films of doped CuPc-TTF and CuPc-TCNQ semiconductors, based on the sequence of glass/ITO/doped semiconductor/Ag. I–V measurements for an area of 3.82 × 10^−4^ cm^2^ in each device were performed to estimate those parameters that quantify the circulation of electric current through the electronic devices. It is worth mentioning that the thickness of the doped-semiconductor layer in this system is the same as the one previously given for the films that were deposited on the Corning glass, quartz, and monocrystalline silicon wafer substrates. Finally, devices with a flat heterojunction structure were manufactured using ITO-coated glass slides (glass-ITO) and ITO-coated polyethylene terephthalate film (PET-ITO) as substrates (Figure 1). Both devices, rigid and flexible, have the following components: (ITO and Ag) electrodes, a selective-hollow-transport layer (CuPc), an electron-transport layer (BCP), and an active heterojunction-plane layer formed by donor species (TTF) and electronic acceptor species (TCNQ). All of them were integrated into the multilayered device glass/ITO/CuPc (20nm)/TTF (20nm)/TCNQ (20nm)/BCP (10nm)/Ag. The active layer has a total area of 4.15 cm^2^ in both devices. For the electrical characterization of the devices, a Keithley 4200-SCS-PK1 (Tektronix Inc., Beaverton, OR, USA) programmable voltage source with auto-ranging pico-ammeter and a Next Robotix (Comercializadora KMox, S.A. de C.V., Benito Juarez, Mexico City, Mexico) sensing station were used. The latter had lighting and temperature controller circuits operating as a solar simulator and emitting electromagnetic radiation in the UV-to-IR wavelength interval.

## 3. Results and Discussion 

### 3.1. Thin Film Structure and Morphology

**Figure 2:**
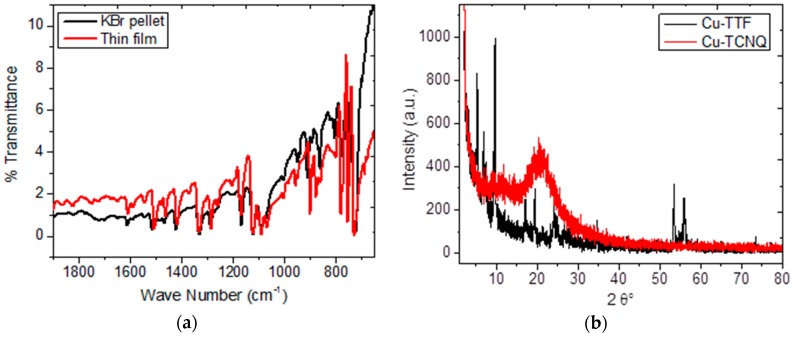
(**a**) IR spectrum of CuPc-TCNQ as KBr pellet and thin film; (**b**) XRD diffractograms of the semiconductor films.

In order to study possible applications of doped organic semiconductors in photovoltaic devices, thin films with these doped semiconductors were prepared using the sublimation technique at low pressures. Given that these organic semiconductors are being considered for use in devices, it is necessary that they exhibit thermal stability during evaporation at low pressures. Semiconductor charge transport increases with temperature in such a way that, to increase device efficiency, it is required that doped semiconductors in the CuPc structure, as well as in TTF and TCNQ dopants, be chemically stable. IR spectroscopy was performed to analyze the main functional groups in CuPc and its dopants, as well as to establish thin-film purity. The presence of impurities related to undesirable reaction products during the doping process may affect the molecular packaging between CuPc and its dopant and thus reduce charge transport within the semiconductor film. The results obtained by FT-IR for the doped semiconductors, in KBr-pellet form as well as deposited on silicon monocrystalline films, can be seen in the Table 1. Representative spectra for CuPc-TCNQ have been collected in Figure 2a. The bands responsible for C=N appear around 1481 and 1334 cm^−1^, while the bands located around 1160, 1119, 779, and 748 cm^−1^ result from the C-H interaction [32,33]. The bands in 1606 and 1094 cm^−1^ result from a C=C stretch within the macrocyclic ring [34]. For CuPc-TTF doped semiconductors, the band related to the C=C interaction is located around 1080 cm^−1^ and the bands corresponding to the C–S bond of the TTF molecule were located at 840 and 780 cm^−1^ [35]. In the CuPc-TCNQ case, there are peaks located around 1204 and 1610 cm^−1^, which are related to C=C–H bending and C=C ring stretch, respectively. These signals are due to the presence of TCNQ in the semiconductor [36]. No major film degradation could be discerned from these results. Similar outcomes were found in both pellet and film semiconductors: such small differences in the measured values as were obtained are mainly due to the concentration of intra and intermolecular stress within the semiconducting material, generated during its evaporation and deposition on the substrate. Another objective sought while performing IR spectroscopy was to detect some impurity traces in the doped semiconductors; however, there was no indication of any chemical by-product of the doping process that could affect molecular packaging. Nevertheless, it is important to mention that, during film deposition, an amorphous structure arose as a consequence of the thermal shock occurring between the semiconductor in the gas phase and the layer at room temperature. The apparition of this structure was verified by XRD. Figure 2b shows the results obtained for both semiconductors, CuPc-TTF and CuPc-TCNQ. According to these results, there is only some degree of crystallinity for the Cu-TTF film and an amorphous structure for the CuPc-TCNQ film. In the Cu-TCNQ case, a broad signal around 23° is observed, which is indicative of the partial amorphous character of this structure [37]. For CuPc-TTF, the diffraction peaks around 17° and 24° correspond to the β phase [38] and the characteristic features of the CuPc polymorphous structure are defined by a single sharp reflection at 2θ = 6.9° [39]. This peak arises from the interlayer spacing of stacks of tilted molecules and is related to the monoclinic α structure of the MPc doped crystals [39]. Based on the above, CuPc-TTF is a mixture of the α-form and the β-form, while CuPc-TCNQ has an amorphous structure.

**Figure 3:**
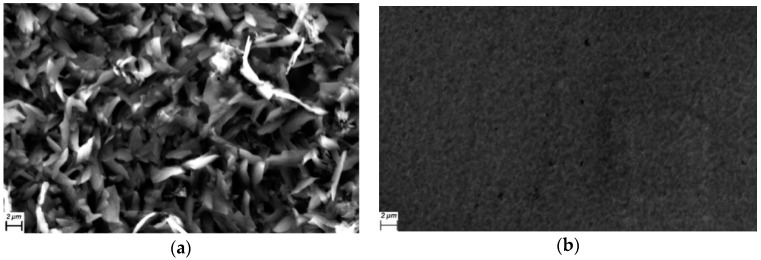
SEM micrographs of (**a**) CuPc-TTF and (**b**) CuPc-TCNQ thin films at 2500×.

The evaporation technique used in this work is a versatile approach used for semiconductor systems in the solid state, and it produces thin, uniform films of acceptable purity for use in optoelectronic devices. In order to evaluate the morphology and structure of the films, SEM was performed over quartz substrates. Figure 3a shows that the CuPc-TTF film has a fibrous-type morphology and, despite the individual structures having a preferential growing direction, or perhaps because of it, the XRD analysis identified some degree of crystallinity, though the whole film was amorphous. Apparently, during its deposition, the first nuclei that formed over the substrate grew in the same direction in which the remaining material ascended in the gaseous state and was deposited on the same nuclei, forming elongated structures as high as 2 μm. It is worth mentioning that films with this morphology hinder electric-charge transport so that, for the manufacture of the devices to be described later, it was considered better to use the planar structure, rather than the disperse heterojunction between the semiconductor and its dopant. In the case of the CuPc-TCNQ film, Figure 3b shows a granular morphology with some heterogeneously-distributed holes. Holes significantly affect charge transport along the film; thus, in this case, it was also advisable to consider a flat or sandwich-type structure in the design of the device.

### 3.2. Thin Film Optical and Electrical Characterization

**Figure 4:**
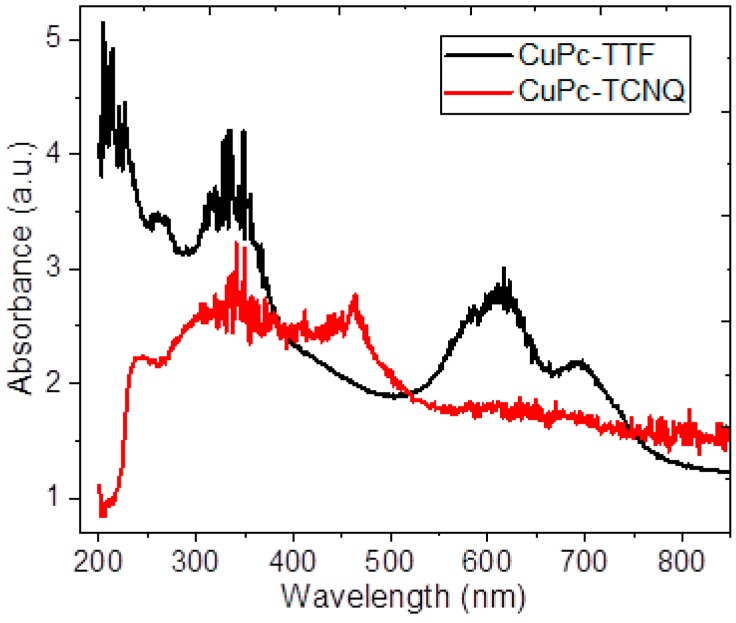
Absorption spectra in the range of 200–900 nm.

**Figure 5:**
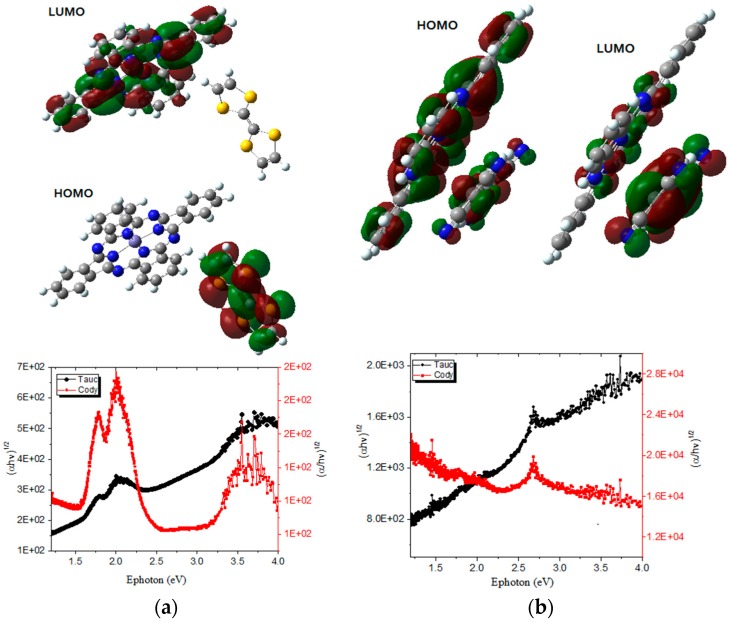
HOMO-LUMO orbitals and Tauc and Cody graphs of the (**a**) CuPc-TTF and (**b**) CuPc-TCNQ films for indirect transitions.

MPcs strongly absorb radiation in the spectrum range between 600 and 700 nm [35,40]. UV–vis spectroscopy allows us to analyze the important electronic transitions in the semiconductor films. Figure 4 shows the absorption spectra of semiconductor films in the wavelength range 200–900 nm; the slope change in both curves indicates that more than one energetic transition occurs between the energy bands of CuPc-TTF and CuPc-TCNQ semiconductors. For the polycrystalline semiconductor CuPc-TTF, photon absorption occurs from the intervention of different optical excitation processes, namely, transitions between separate levels with higher energies than those of the bandgap in the ultraviolet region, and is due to transitions from the upper edge of the conduction band. Moreover, the CuPc-TTF spectrum clearly shows the presence of MPc: the B-band in the near UV region, and the Q-band on the red side of the spectrum. These bands are related to the molecular orbitals of the aromatic system with 18π-electrons and to the overlapping orbital on the central metallic atom [40]. For CuPc-TTF, two peaks can be observed: a high energy peak around 613 nm, and a second one, a low energy peak, around 695 nm. Such two peaks correspond to the Q-band transition of the CuPcs, assigned to the first π-π* transition of the Pc macrocycle [40]. The broadening of the absorption bands can be related to the aggregation of molecules in the thin film [40]. From the spectra, it can also be seen that the CuPc-TTF has its B-band around 330 nm [35,40]. The electronic π-π* transition corresponds to a B-band, which gives the fundamental absorption edge; the B-band is due to a_2u_ (π) → e_g_ (π*) and b_2u_ (π) → e_g_ (π*) transitions [41,42]. Figure 4 shows that the B-band of the CuPc-TCNQ semiconductor is quite similar to that of CuPc-TTF, the most important difference being the displacement of the two curves due to the dopant change. It is important to notice that CuPc-TCNQ does not include a Q-band in its spectrum, which suggests less charge transport in this semiconductor, which is accentuated by the amorphous character of its film. Thus, a forbidden band, or bandgap, between the HOMO (highest occupied molecular orbital) and the LUMO (lowest unoccupied molecular orbital), as shown in Figure 5 would be expected to be larger than that of the CuPc-TTF semiconductor, with the effect of TTF as an electronic donor of the CuPc molecule predominating over the electronic acceptor effect of the TCNQ facing the macrocycle. This can be proved through the bandgap calculation.

The optical bandgap is a fundamental parameter to consider in the manufacture of photovoltaic devices, as the measurements of spectral variations of band-band transitions can be used to determine the bandgap in the semiconductor. The electronic properties of semiconductors, such as light absorption efficiency, are determined by the bandgap. The Tauc model of amorphous semiconductors is one of the most used models for bandgap calculations. This model is used to calculate the optical bandgap for direct electronic transitions (αhν)^2^, as well as for indirect electronic transitions (αhν)^1/2^ [43,44]. The distinction concerns the relative positions of the conduction band minimum and the valence band maximum. In a material with direct bandgap, both extremes occur in the central wavenumber zone and, during the transition, the electron jumps from the low-energy (valence) band to the high-energy (conduction) band by absorbing a photon. In an indirect bandgap material, the conduction band minimum is not located in the central zone, but in an edge area or next to it. In this case, it is not possible during a transition for an electron to leap between the valence and conduction bands by only absorbing a photon, as the transition should also involve a phonon for momentum preservation [45]. The Tauc model considers that the variation in the absorption coefficient (α) depends on the photon energy (*E_photon_*). The absorption coefficient (α) is obtained by means of [43,44]

(1) $$\alpha =-\ln\left(\frac{T}{d}\right)$$

where *T* is the percentage transmittance obtained by UV–vis spectroscopy and *d* is the thickness of the film. The photon energy is obtained through

(2) $$E_{photon}\left(eV\right)=\frac{hc}{\lambda }$$

where *h* is Planck constant, *c* is the speed of light in vacuum, and λ is the wavelength.

Subsequently, we obtain a graph of (*αhν*)^n^ vs. *E_photon_*. The absorption parameter of interest is (*αhν*)^n^, with n = 2 for direct transitions and n = 1/2 for indirect transitions. From the resulting graph (see Figure 5 for indirect transitions), the linear zone is located, a tangent line is drawn and the point of intersection with the *E_photon_* axis is obtained. The intersection point corresponds to the bandgap. The optical bandgap values are shown in Table 2 and, as was expected from the UV–vis spectra, the lower bandgap values correspond to the CuPc-TTF semiconductor. It is worth mentioning that the dominant transitions for this semiconductor are of a direct nature related to their polycrystalline character, while indirect transitions occur in the CuPc-TCNQ semiconductor, related to its amorphous character and lower bandgap. The crystalline-like structure found in CuPc-TTF is a desirable feature of thin films made of doped semiconductors for photovoltaic applications, in order to create preferential pathways for electric charge transport and to favor direct transitions between electrons jumping from the HOMO orbital to the LUMO orbital. Theoretical calculations by means of the DFT method were carried out in order to obtain a reliable description of the frontier molecular orbital region. These functions are shown in Figure 5; in the case of CuPc-TTF (Figure 5a), the HOMO orbital is located in TTF, while the LUMO is located in CuPc. Thus, a considerable electronic flow is expected from TTF to CuPc. In the case of the CuPc-TCNQ (Figure 5b), the isosurfaces calculated for the molecular orbitals HOMO and LUMO show that the electronic density in the semiconductor is distributed throughout the π-conjugated skeleton. In order to verify the bandgap obtained through the Tauc method and determine the influence of thickness in the bandgap of the sandwich-like structures, an analysis based on the Cody model was also performed. Cody et al. [44,46] hypothesize that the bandgap behavior is related to a fundamental curvature in the spectral dependence of (*αhυ*)^n^ = *f*(*E_photon_*), which they hold responsible for the increases in the Tauc optical gap as related to thickness [43]. According to the Cody model [44,46], the semiconductor-film bandgap should be determined from extrapolation of the linear trend observed in the spectral dependence of (α/*h*ν)^n^ vs. *E_photon_* over a limited range of photon energies. Figure 5 shows the Tauc and Cody curves for indirect transitions. The abscissa-axis intercept of this linear extrapolation corresponds to the limit of the Cody bandgap as thickness becomes negligible. Considering that the bandgap in organic semiconductors varies between 1.5 and 4.0 eV, the results obtained through the Cody model do not show significant changes with respect to those obtained through the Tauc model. The above results suggest that the thickness of these materials does not have a significant effect on charge transport within the semiconductor films. Thus, the bandgap can be calculated by any of the two semi-empirical models to determine the expected behavior of multilayer devices manufactured from these semiconductors. Finally, in order to verify the experimental values of the bandgap obtained by the Tauc and Cody methods, the theoretical bandgap was obtained by means of the DFT method. The results are shown in Table 2 and, in accordance with the experimental determination, CuPc-TTF is the material with the lowest bandgap. The difference between the experimental and the theoretical values is mainly due to the limits given by the precision of experimental data and the computational errors of the DFT method [47]. Nevertheless, both results firmly put the bandgap for the materials studied below 4.0 eV, which is considered the limiting bandgap for organic semiconductors [47]. It is also necessary to consider that the bandgap also depends on structural factors of the material, such as alternations in bond length, presence of impurities resulting from the synthesis process, and degree of stacking of the electron donor and acceptor molecules.

**Figure 6:**
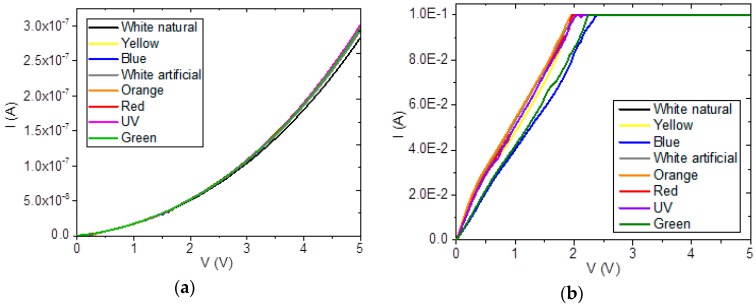
I–V- behavior in (**a**) CuPc-TTF and (**b**) CuPc-TCNQ films.

Simple devices with the structure glass/ITO/doped semiconductor/Ag were prepared and I–V measurements were performed to estimate those parameters that quantify current flow through the devices. The goals of the measurements and subsequent analysis were to determine if doped CuPc could work as a semiconducting material and to find the effects of the different dopants on its behavior. This study also had the objective of evaluating the donor behavior of the TTF and the acceptor behavior of the TCNQ with respect to CuPc, which is known by its efficiency as anodic layer and hole transporter in optoelectronic- and photovoltaic-type devices [13,22,23,24,25,26,27]. I–V measurements for each device are shown in Figure 6. The devices were prepared with a bulk heterojunction structure, with the active film formed by a species of heterogeneous mixture between CuPc and its dopant. The compounds were deposited together to provide a random distribution and a higher contact surface that could allow higher excitonic diffusion. In Figure 6a, it is observed that the CuPc-TTF device shows a behavior similar to that of a directly-polarized rectifier diode, while the device with an active layer of CuPc-TCNQ (Figure 6b) shows an ohmic-like behavior in the range up to about 1 to 1.6 V, with a saturation effect at larger voltages. This device transports the largest amount of electric current, around 0.1 A; despite its being a bulk heterojunction, it does not have well-defined regions for the selective transport of charge carriers towards the right electrode, and its behavior changes from ohmic to insulating (see Figure 6b). Furthermore, the behavior of CuPc-TCNQ can also be affected by the fact that, as has been demonstrated elsewhere [25,27], doping p-type semiconductors such as MPc with TCNQ can turn them into n-type semiconductors [25,27]. Regarding CuPc-TTF, when a voltage is applied, the depletion zone becomes narrower and the current intensity increases. The depletion, or space-charge, zone is devoid of carriers as a result of charge transfer at the electrode-semiconductor interface. The diode-like behavior of this material could be attributed to the addition of TTF in the doping process, which leads to the formation of a homogeneous material with an apparent *p-n* junction. CuPc-TTF behavior could be attributed to the formation of alternative paths for carrier conduction due to p-type doping of the organic semiconductor with TTF, as has been reported in earlier studies [48]. It is worth mentioning that the measurements were done at room temperature under different illumination conditions in order to evaluate how would the device behave under the effect of solar irradiation, including natural and artificial white light, as well as red, orange, yellow, green, blue, and UV light. In the device with CuPc-TTF, there are no observed differences in electrical behavior with color changes. On the other hand, ultraviolet radiation increases electric current at lower voltages in the CuPc-TCNQ device. In both cases, the ITO layer is included to provide an electrical contact that permits the electrical properties of the doped semiconductors to be tested. From the above observations, it can be established that both materials behave as semiconductors. This fact is also supported by the bandgap values of 1.1 eV (direct transitions) for CuPc-TTF and 1.6 eV (indirect transitions) for CuPc-TCNQ, which are close to those of silicon (1.1 eV). For a semiconductor film to be used in optoelectronic devices, a bandgap smaller than 1.8 eV would typically be expected [47].

### 3.3. Electric Characterization of Heterojunction Planar Devices

**Figure 7:**
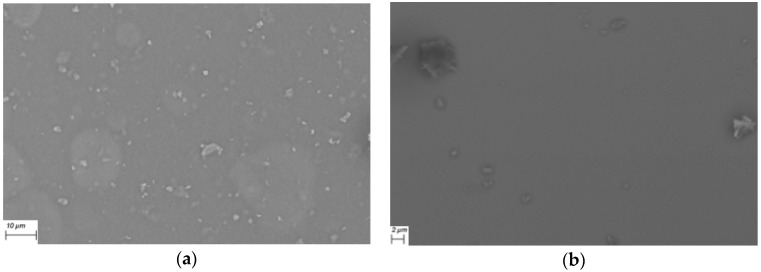

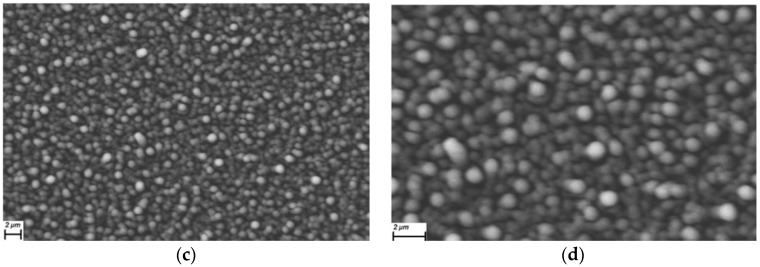
SEM micrographs of films from devices using (**a**) glass at 2500×, (**b**) glass at 5000×, (**c**) PET at 2500×, and (**d**) PET at 5000×.

Given that both films behave as organic semiconductors, two devices with a flat heterojunction structure were built in order to better understand the donor/acceptor behavior of the dopants, this time as components of the devices’ active layer and also to analyze the effect of substituting the glass substrate with PET, which has been proposed as an alternative substrate for devices (see Figure 1). The adherence to the substrate will promote efficient charge transport within the device, which is a worthy reason for studying the effect of glass and PET on the devices’ electric behavior. Taking into consideration the irregular and heterogeneous morphology of the films produced from doped semiconductors by disperse heterojunction, the two devices had a sandwich-type structure, as shown in Figure 1: (glass/ITO/CuPc/TTF/TCNQ/BCP/Ag). It is important to emphasize that the fabricated devices have not been optimized, that is, the effect of thickness has not been taken into account, as the purpose of the study is to analyze the use of different types of substrates, as well as evaluate the performance of TTF and TCNQ in the active layer. The active layer is responsible for radiation absorption and is where photocurrent arises. TTF is the photoactive compound responsible for radiation absorption and charge-carrier transport through the HOMO states. TCNQ transfers electrons to the cathode through its low-energy LUMOs. The introduction of CuPc and BCP interfacial layers in the photovoltaic devices permits the selective extraction of charge carriers from the active layer and prevents those charges generated in the active layer from moving in an undesirable direction. These selective transport layers protect the active layer from physical and chemical interactions with the electrodes and determine the relative polarity of the device. Efficient charge transport inside a device occurs when charges can easily move from one molecule to another and are not trapped or dispersed. However, charge mobility within the device is affected by such factors as thin-film quality. An adequate characterization of the sandwich structures’ morphology requires the surface to be uniform and free of impurities, so that the electric charge transport be efficient. SEM was performed on glass and PET substrates in order to analyze the morphology of the films’ structures. From the images shown in Figure 7, it is clear that they have different surface morphologies. As shown in Figure 7a, the device on glass shows different phases related to the layers that integrate it: on one hand, rounded particles of sizes greater than 10 μm, with other particles of different morphology which, according to Figure 7b, may be smaller than 2 μm. It is also observed that these particles are located over a homogeneous film. The deposition of the films took place at different stages: initially, the first compound was deposited directly over the substrate at room temperature, forming a homogeneous layer. Afterwards, the second compound came into contact with the previously-deposited layer, which was above room temperature. Due to the thermal gradient that occurred during the deposition, additional nuclei were formed and slowly grew into grains of a considerable size. The images shown in Figure 7c,d, corresponding to the device deposited on PET, are in remarkable contrast with those deposited on glass. In the PET case, round particles with sizes of around 1 μm are uniformly distributed above the substrate. A homogeneous nucleation seems to have occurred in this case, with nuclei arising from their own constituents, unlike the heterogeneous nucleation that seems to have occurred in the glass case. This suggests that the previously-deposited film served as a nucleating layer; the first layer, deposited over glass, was affected by the substrate and each nucleus within it grew according to preferential directions. Some larger structures, defined by elongated particles which grew in different directions—such as those generated in heterogeneous-type nucleation—can be observed in Figure 7b. It is important to mention that CuPc deposits directly over the ITO film, both in the device built on glass and in the one built on PET. However, this film has different characteristics when it deposits on materials of such a different nature as are ceramics (in the case of glass) and polymers (in the case of PET). According to Porai-Koshits and Vogel [49,50,51], glass does not have a homogeneous distribution, but rather a grid-like structure formed by isolated, highly-ordered regions that are joined to the surrounding, disordered vitreous matrix. In very transparent and optically homogeneous glasses, micro-heterogeneities are produced as a consequence of the alternation of completely disordered and partially ordered regions, according to Vogel [50,51]. This leads to an ITO-deposited film not having the same surface characteristics as a film of the same material deposited on PET. Unlike glass, PET is formed by molecular chains in which polarity is the relevant feature. The chemical structures having the largest influence on PET molecular chains are polar aromatic groups; these groups based on benzene rings provide rigidity and promote homogeneous crystallization [52,53].

**Figure 8:**
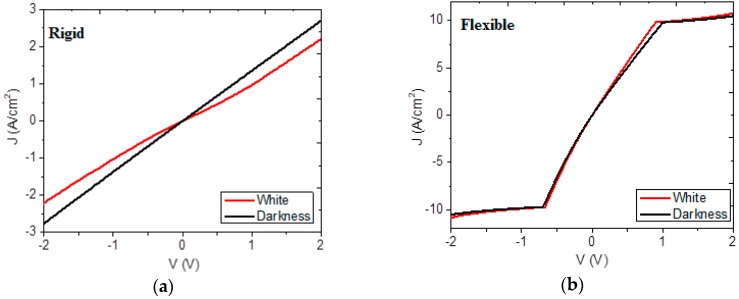
J–V behavior in (**a**) rigid and (**b**) flexible devices.

When solar radiation hits the semiconductor materials in the devices, it produces electron–hole bound states, called excitons. Exciton dissociation leads to the generation of free charge carriers that migrate from the TTF-TCNQ active layer, where light absorption takes place, passing through selective layers of CuPc and BCP, into the electrodes where the free carriers are collected. Given that, as observed in Figure 6, the different types of electromagnetic radiation incident on semiconductors produce similar effects, the devices were electrically characterized at room temperature in the presence of white light and in darkness. From the I–V measurements, current density J and its dependence on applied voltage in rigid-glass and flexible-PET substrates was determined and is shown in Figure 8. As expected, given the morphology information provided in Figure 7, the electric behavior is significantly different between the device deposited over the rigid substrate and the one deposited over the flexible substrate. Figure 8a shows the J–V curve, which is typical of a semiconductor. The rigid device does not show any variations between the forward and reverse operation zones, and a small variation when exposed to illumination or left in darkness. Maximum current density values in forward and reverse operations are of around 3 and −3 A/cm^2^, respectively. In general, an ohmic behavior appears throughout the J–V curve with no special features, which suggests possible optoelectronic applications where a stable, layered semiconductor is required. Thus, according to Figure 8a, the combination of TTF and TCNQ could allow the fabrication of ambipolar devices, such as organic light-emitting transistors [47], with both p-type and n-type behaviors in its active layer. Figure 8b shows two regimes in the curve corresponding to the flexible device: one corresponding to ohmic conduction at voltages lower than 1 V, and another that corresponds to space charge limited current (SCLC) at higher voltages and is related to an exponential trap distribution [28,54,55,56]. From the above, the rigid device and the flexible device up to 1 V (before saturation) are described by an ohmic law of the form [28]

*J* = *ep*_0_*μ*(*V*/*d*) (3)

where *p*_0_ is the concentration of the thermally generated holes, *e* is the electronic charge, *μ* is the hole mobility, *V* is the applied voltage, and *d* is the film thickness. Electric transport properties in the devices are associated with a hopping model, due to the difference between the HOMO and LUMO energy levels and the varying work function levels of the electrodes. In the ohmic-contact case of the flexible device, spatial charges are formed in the vicinity of the electrodes, which oppose current flow through the flexible device. For a sufficiently large applied voltage (1 V), current saturation occurs. In this SCLC model for annealed devices, *J* is given by the expression [54,55]

*J* = *N*_v_*eμ*(*ε*_r_/e*P*_0_*kT*_L_)*^l^* (*V^l^*^+1^/*d*^2*l*+1^) (4)

In addition to the previously defined symbols, *N*_v_ is the effective density of states in the valence band, *ε*_r_ is the permittivity, *P*_0_ is the concentration of traps per unit of energy, *k* is Boltzmann constant, *T*_L_ is a temperature parameter which characterizes the trap distribution and

*l* = *T*_L_/*T* (5)

*T* is the temperature of the device and *l* is the slope of the ohmic regime. The total trap concentration *N_t(e)_* is equal to

*N_t(e)_* = *P*_0_*kT*_L_ (6)

For the flexible device, current density values can be as high as 10 A/cm^2^. Thus, even when it reaches saturation at lower voltage values, this material could be employed as a semiconductor in applications where higher current density levels are required. According to Figure 8b, the TTF-TCNQ combination could also be used as active layer in ambipolar transistors that will feature hole accumulation at negative voltages and electron accumulation at positive voltages [47,57]. Regarding the electrical behavior of the devices, the calculated values of the parameters derived from Equation (3) for the ohmic region of the two devices and Equation (4) for the SCLC region of the PET device are shown in Table 3. Considering an ohmic contact between the semiconductor and the electrodes, these values seem to imply that, if the injection and/or extraction energy barriers are neglected and one assumes the absence of traps and neglects the disorder inherent to such materials, charge mobility in the flexible device could be estimated by following the SCLC method and comparing the results obtained with those widely studied for CuPc. This phthalocyanine is recognized as a p-type semiconductor, and its hole mobility (*μ*) obtained by the SCLC method in the PET device depends on its relationship with TTF, TCNQ, and BCP, as it only acts as a hole carrier layer. The μ value for the device is inferior to those within the interval reported for CuPc (10^−8^–10^−7^ m^2^·V^−1^·s^−1^) [55,58,59]. With respect to the results obtained for the concentration of thermally generated holes (*p*_0_) in the devices, they are superior by nearly five orders of magnitude, respectively, to those found for the CuPc films [58]. With respect to the concentration of traps per unit of energy (*P*_0_) and total trap concentration (*N_t(e)_*), the values obtained for the relevant coefficients are within the reported ranges for some MPcs: 8 × 10^43^ to 1.15 × 10^47^ J^−1^·m^−3^ and 6 × 10^20^ to 9.3 × 10^26^ m^−3^, respectively [28,47,54,55,56,57]. The differences in the J–V behavior of each device, as well as the differences found in charge mobility, could be associated to the substrates used in the manufacture of the device. PET not only generates greater adherence among the different layers that integrate the device, but also promotes a homogeneous morphology, which favors electric transport along the device, especially under lighting conditions. Regarding the structure studied by SEM for both devices (see Figure 7), charge transport could be explained by a hopping model [28]. Since the different layers which integrate devices manufactured by a flat union may have structural variations, charge transport is produced through electronic transfers between energy levels located in spatially-close molecules [60]. If there is a considerable degree of disorder within the film, the energy-level distribution can be affected in such a way that energy levels beyond the edges of the valence or conduction bands could appear, hindering carrier circulation. Carrier immobilization would prevent them from contributing to charge transport. It seems that device behavior is conditioned by morphology, rather than by the strength of the intermolecular interactions of the films in the devices. Nevertheless, further work is still required in this regard involving, among other things, the characterization of the electrical behavior of optimized devices.

## 4. Conclusions and Further Work

Using simple synthesis, the doping by disperse heterojunction of organic semiconductors was carried out in order to evaluate the optical and electrical behaviors of the TTF donor and the TCNQ acceptor of a CuPc semiconductor. The semiconductor films were deposited on different substrates by vacuum thermal sublimation, which, according to the results obtained by IR spectroscopy, is an adequate technique, not only to produce high-purity thin films, but also to manufacture optoelectronic devices without chemical decomposition of the organic semiconductors that integrate them. The optical properties of the doped films were analyzed by UV–vis spectroscopy and it was found that, although their bandgap is in the range of organic semiconductors, according to the Tauc and Cody models, it is independent of the semiconductor-film thickness. The experimental bandgaps were compared with those obtained theoretically with DFT method and both results locate the materials studied, within the range of the organic semiconductors. From the electrical characterization of the thin films, it was found that their electric behavior is ohmic when the semiconductor is doped with TTF, while it is rectifying-like when doped with TCNQ. This is so because of the strong donor and acceptor character, respectively, of each dopant. TTF behaves as a p-type organic molecular semiconductor (hole carriers), whereas TCNQ behaves as an n-type molecular semiconductor (electron carrier). These two compounds integrated the active layers of photovoltaic devices with flat heterojunction structures, built over rigid and flexible substrates in order to evaluate the use of organic semiconductors in flexible microelectronics and to study their optoelectronic behavior. A higher charge transport was achieved by the device manufactured on a flexible substrate than by the one on the rigid substrate. This suggests the possibility of including flexible devices in applications where high mechanical stress could fracture glass substrates. On the other hand, the use of PET, with a high elastic module, could allow charge absorption through its polymer chains. The active layer formed by TTF and TCNQ had an ambipolar behavior, with balanced hole and electron mobilities. In any case, it is advisable to carry further work in order to optimize these devices regarding structural layer ordering, perhaps by recrystallization annealing and temperature adjustments during film deposition. Layer thickness is another criterion requiring optimization, and perhaps the same could be argued about the introduction of additional layers capable of injecting holes as well as electrons. Finally, it may prove useful to reconsider device structure and, in addition to the conventional architecture, evaluate the feasibility of inverted (p-i-n) or tandem architectures. All of the above may contribute to improving electronic parameters such as current density and charge mobility.

## Figures and Tables

**Table 1:** Characteristic FT-IR signals for doped pellet and film semiconductors.

Sample	CuPc ν(C=N) cm^−1^	CuPc ν(C–H) cm^−1^	CuPc ν(C=C) cm^−1^	TTF ν(C=C) cm^−1^	TTF ν(C–S) cm^−1^	TCNQ ν(C=C–H) cm^−1^	TCNQ ν(C=C) cm^−1^
*CuPc-TTF* (KBr pellet)	1479, 1334	1164, 1121, 780, 752	1610, 1101	1089	846, 781	-	-
*CuPc-TTF* (film)	1484, 1335	1167, 1121, 778, 751	1597, 1094	1081	844, 780	-	-
*CuPc-TCNQ* (KBr pellet)	1483, 1333	1167, 1115, 774, 752	1609, 1100	-	-	1203	1610
*CuPc-TCNQ* (film)	1481, 1332	1165, 1120, 772, 750	1610, 1093	-	-	1206	1610

**Table 2:** Theoretical and experimental bandgaps.

Sample	Tauc Direct Bandgap (eV)	Tauc Indirect Bandgap (eV)	Cody Direct Bandgap (eV)	Cody Indirect Bandgap (eV)	DFT Bandgap (eV)
CuPc-TTF	1.1	1.4	1.7	1.6	2.59
CuPc-TCNQ	2.2	1.9	2.3	2.2	2.73

**Table 3:** Electrical properties for rigid and flexible devices in darkness and under illumination

Parameters, Darkness			Parameters, Illuminated		
μ (glass)	1.59 × 10^−9^	(cm^2^)/V·s	μ (glass)	3.94 × 10^−10^	(cm^2^)/V·s
p_0_ (glass)	7.74 × 10^23^	m^−3^	p_0_ (glass)	7.74 × 10^23^	m^−3^
P_0_ (glass)	-	1/(J·m^3^)	P_0_ (glass)	-	1/(J·m^3^)
Nte (glass)	-	1/m^3^	Nte (glass)	-	1/m^3^
μ (PET)	1.84 × 10^−9^	(cm^2^)/V s	μ (PET)	4.51 × 10^−9^	(cm^2^)/V s
p_0_ (PET)	7.74 × 10^23^	m^−3^	p_0_ (PET)	7.74 × 10^23^	m^−3^
P_0_ (PET)	5.58 × 104^3^	1/(J·m^3^)	P_0_ (PET)	6.05 × 10^43^	1/(J·m^3^)
Nte (PET)	1.22 × 10^24^	1/m^3^	Nte (PET)	1.25 × 10^24^	1/m^3^
